# Association between preoperative chronic steroid use and pulmonary embolism in adult tumor craniotomy: Linking role of platelet-to-white blood cell ratio

**DOI:** 10.1371/journal.pone.0355416

**Published:** 2026-08-04

**Authors:** Bo Gong, Shenshen Liu, Shaomin Chen, Lei Yu, Lei Liu, Li Tan

**Affiliations:** 1 Department of Emergency Medicine, General Hospital of Central Theater Command, Wuhan, Hubei, China; 2 Department of Disease Control and Prevention, General Hospital of Central Theater Command, Wuhan, Hubei, China; 3 Department of Transfusion Medicine, General Hospital of Central Theater Command, Wuhan, Hubei, China; Hokkaido University: Hokkaido Daigaku, JAPAN

## Abstract

Previous studies indicate that preoperative chronic steroid use is associated with an increased risk of postoperative pulmonary embolism (PE) in adult patients undergoing tumor craniotomy. We aimed to evaluate whether the platelet-to-white blood cell (PLT/WBC) ratio may partially explain the association. This retrospective study involved a secondary analysis of data from the American College of Surgeons National Surgical Quality Improvement Program database, encompassing 15,274 patients who underwent tumor craniotomy. Multivariable logistic regression was performed to assess the association between preoperative chronic steroid use and 30-day postoperative PE. Furthermore, a mediation analysis was performed to explore the role of the PLT/WBC ratio in this association. Preoperative chronic steroid use was present in 15.18% (n = 2,319) of patients, and the rate of postoperative PE was 1.41% (n = 215), with a higher rate in chronic steroid users compared to non-users (2.37% vs. 1.24%). Multivariable analysis revealed a positive association between preoperative chronic steroid use and postoperative PE (OR = 1.90, 95% CI: 1.24, 2.90). Mediation analysis demonstrated that the PLT/WBC ratio significantly explained part of the association between chronic steroid use and postoperative PE (*P* = 0.006). Our findings further supported the association between preoperative chronic steroid use and elevated odds of postoperative PE in adults undergoing tumor craniotomy. Additionally, our analysis revealed that preoperative PLT/WBC ratio potentially acts as a partial linking factor in this association. Future studies are required to substantiate these findings.

## Introduction

Surgical resection remains the principal therapeutic strategy for adult intracranial tumors [1], yet it is associated with significant perioperative risks [2]. Among these complications, postoperative pulmonary embolism (PE) is of particular concern due to the hypercoagulable state inherent to malignancy and postoperative immobilization [3,4]. PE represents a severe manifestation of venous thromboembolism (VTE) and serves as a potentially fatal postoperative complication [5,6]. The occurrence of this complication leads to significantly worse overall clinical outcomes in patients with brain tumors [7,8]. Therefore, exploring preoperative factors associated with PE is essential for improving the understanding of postoperative complication risks in this population.

Corticosteroids are frequently administered preoperatively to patients with brain tumors, primarily to mitigate peritumoral edema or manage concurrent chronic conditions [9–11]. Importantly, in the context of tumor craniotomy, chronic steroid use is not solely a pharmacologic exposure. It also frequently serves as a clinical proxy of greater underlying disease severity, as patients requiring prolonged steroid therapy often present with more extensive peritumoral edema, higher-grade tumor biology, metastatic burden, or impaired functional status [12,13]. Despite these therapeutic applications, prolonged steroid exposure is known to exert systemic physiological effects, notably impacting metabolic functions and hemostatic balance [14–16]. Consistent with these observations, studies utilizing large surgical registries have reported a positive association between preoperative chronic steroid use and the occurrence of postoperative PE in neurosurgical patients, including those undergoing craniotomy [17–19]. While this clinical association is increasingly recognized, the potential factors underlying this relationship remain not fully explored. Moreover, disentangling the pharmacologic effects of steroids from the confounding influence of the clinical indications for their use is inherently challenging in observational registries that lack detailed data on steroid dosing, indication, tumor characteristics, and perioperative management [20,21], a limitation that should be considered when interpreting findings from studies of this nature. Notwithstanding this inherent complexity, investigating the potential linking factors involved in this association is crucial for better understanding the relationship between chronic steroid use and PE in this population.

As part of its systemic physiological effects, corticosteroid exposure is known to alter hematological profiles, notably by inducing leukocytosis and modulating platelet counts [22–24]. Both white blood cells (WBCs) and platelets play pivotal roles in the pathogenesis of VTE, acting as key drivers in the interaction between inflammation and coagulation [25,26]. Moreover, recent evidence demonstrates that the PLT/WBC ratio is significantly associated with adverse clinical outcomes in other thrombo-inflammatory vascular diseases, potentially reflecting the severity of these hypercoagulable states [27–29]. Given these observations, the PLT/WBC ratio represents a biologically plausible linking factor that may connect the systemic effects of chronic steroid use to the increased odds of postoperative PE in this population.

Notwithstanding these biological explanations, the specific linking role of the PLT/WBC ratio in the relationship between preoperative steroid exposure and postoperative PE has not been empirically evaluated. We hypothesized that preoperative chronic steroid use is associated with increased odds of postoperative PE in adult patients undergoing craniotomy for tumors, and that the PLT/WBC ratio may play a linking role in this association. Accordingly, the present study utilized the American College of Surgeons National Surgical Quality Improvement Program (ACS-NSQIP) database to investigate the association between preoperative chronic steroid use and 30-day postoperative PE in this specific population, and explicitly explored the potential linking role of the PLT/WBC ratio in this association.

## Methods

### Dataset source

Data for this retrospective cohort study were directly retrieved from the publicly available supplementary material of a previously published investigation [30]. While the primary data in that study was originally sourced from the ACS-NSQIP database spanning the years 2012–2015, our analysis exclusively utilized the extracted dataset published by those authors. The dataset contains clinical records of 18,642 adult patients undergoing craniotomy for brain tumor resection across approximately 400 U.S. academic and community medical centers. As this supplementary dataset is distributed under the Creative Commons Attribution License, which permits open-access reuse and secondary analysis with proper citation, our study was conducted in full compliance with copyright regulations.

### Study participants

**Fig 1:**
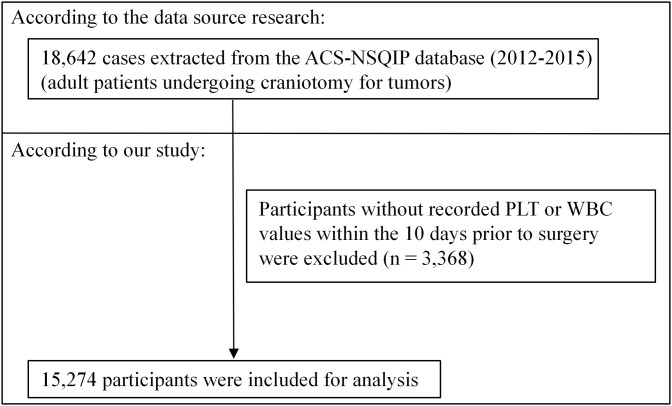
Flowchart illustrating the process of participant exclusion. ACS-NSQIP, American College of Surgeons National Surgical Quality Improvement Program; PLT, platelet; WBC, white blood cell.

The original dataset, comprising 18,642 adult patients, served as the source population for this study. We began by assessing data completeness for the exposure and outcome variables. In the pre-cleaned secondary dataset provided by the original authors, the exposure (preoperative chronic steroid use) was recorded under the variable STEROID (coded as “Yes” or “No”), and the outcome (postoperative PE) was recorded under the variable PULEMBOL (coded as “No Complication” or “Pulmonary Embolism”). Notably, there were no missing, unknown, or null values for these specific variables in the secondary dataset. The days-to-event variable (DPULEMBOL) was not included in this secondary dataset. To facilitate the computation of PLT/WBC ratio in proximity to the surgical procedure, the study population was restricted to individuals with recorded platelet and WBC measurements within 10 days prior to surgery. As a result, 3,368 patients were excluded due to missing platelet or WBC values, yielding a final analytical cohort of 15,274 participants (Fig 1).

### Ethics declarations

This study represents a secondary analysis of a publicly available, de-identified dataset originally derived from the ACS-NSQIP database [31]. As the dataset contains no personally identifiable information, this study was exempt from Institutional Review Board review and the requirement for informed consent. The study was conducted in accordance with the Declaration of Helsinki.

### Assessment of exposure and linking factor

The exposure of interest in this study was preoperative chronic steroid use. In alignment with the definitions provided in the ACS-NSQIP user guide and corroborated by existing literature [21,32], this variable was defined as the regular administration of oral or parenteral corticosteroids for a chronic medical condition within the 30 days preceding surgery. The definition explicitly excluded corticosteroids administered through topical, inhalation, or rectal routes. Additionally, patients who received only a short-term course (10 days or fewer) or a single pulse of corticosteroids were not classified as chronic users. Notably, in the context of brain tumor patients, this variable may capture both corticosteroids prescribed for chronic conditions (e.g., chronic obstructive pulmonary disease, asthma, rheumatologic disease) and dexamethasone administered for persistent peritumoral edema management, provided the duration exceeded 10 days. However, the ACS-NSQIP database does not contain additional variables that specify the precise indication for steroid therapy, precluding differentiation between these clinical scenarios. Based on these criteria, the study cohort was stratified into two groups: the chronic steroid group and the non-steroid group.

The preoperative PLT/WBC ratio was employed as the linking factor in our analysis. This variable was calculated as the ratio of platelet count to WBC count, with both parameters expressed in units of 10^9^/L.

The temporal framework of this study was designed to support a plausible chronological sequence between the exposure and the linking factor. Because chronic steroid use required regular corticosteroid administration for more than 10 consecutive days within the 30 days preceding surgery, the period of steroid exposure would have preceded or at minimum overlapped with the PLT/WBC ratio measurement window (restricted to within 10 days prior to surgery). This design feature was intended to enhance the plausibility that the measured PLT/WBC ratio captures hematological changes occurring in the context of, or coinciding with, the period of chronic steroid exposure.

### Assessment of outcome

The outcome of interest in this study was the occurrence of PE within 30 days after craniotomy. As detailed in the ACS-NSQIP user guide and consistent with previous studies [33,34], this outcome was systematically determined by trained clinical reviewers at each participating institution through rigorous chart review. Briefly, a PE event was defined as the lodging of a blood clot in the pulmonary artery with subsequent obstruction of blood supply to the lung parenchyma. Following the ACS-NSQIP technical specifications, because preoperative imaging is not routinely available to prove the absence of a prior clot, the variable requires a “new diagnosis” rather than definitive proof of postoperative onset, meaning the thrombus was not previously known. To qualify as a postoperative complication, this new diagnosis must have been established within the 30-day postoperative window and confirmed via definitive imaging modalities (e.g., CT angiogram, V-Q scan, pulmonary arteriogram, or transesophageal echocardiography).

### Other covariates

We included covariates based on clinical relevance and existing literature [17,18,35,36]. Demographic characteristics consisted of sex, race (White, Black, or Other), and age range (stratified as 18–40, 41–60, 61–80, and ≥ 81 years). Clinical and comorbidity profiles covered smoking status, body mass index (BMI) categories (< 25.0, 25.0–29.9, and ≥ 30.0 kg/m²), diabetes, hypertension, disseminated cancer, and bleeding disorders. Regarding patient status, variables included functional health status (independent vs. dependent), emergency case status, and American Society of Anesthesiologists (ASA) classification (grouped as Class I-II vs. Class III-V). Preoperative sepsis was defined as a binary variable indicating the presence of systemic inflammatory response syndrome, sepsis, or septic shock. Finally, laboratory parameters comprised serum sodium, albumin (ALB), blood urea nitrogen (BUN), and aspartate aminotransferase (AST).

### Statistical analysis

The distribution of continuous variables was assessed using histograms. Normally distributed continuous variables were reported as mean ± standard deviation (SD) and compared using the t-test, while skewed data were described as median (interquartile range [IQR]) and analyzed via the Mann-Whitney U test. Categorical variables were summarized as frequencies with percentages and compared between groups using the Chi-square test.

To examine the relationship between preoperative chronic steroid use and postoperative PE, we employed multivariable logistic regression models. The findings are presented as odds ratios (ORs) accompanied by 95% confidence intervals (CIs). Covariates were chosen for adjustment based on their clinical significance, their association with the outcome, or if they caused a change in effect estimates exceeding 10% [37]. As a result, the final models were adjusted to account for variables such as age range, sex, race, smoking status, BMI, diabetes, hypertension, disseminated cancer, bleeding disorders, preoperative sepsis, emergency case, functional health status, ASA classification, BUN, AST, sodium, and ALB. Additionally, stratified analyses were conducted employing stratified logistic models to ensure the consistency of the association across various subgroups. Interaction effects were evaluated through likelihood ratio tests, comparing models with and without interaction terms.

Furthermore, a multivariable linear regression analysis was conducted to investigate the relationship between preoperative chronic steroid use and the linking factor (PLT/WBC ratio). Model diagnostics, including the Normal Q-Q plot of standardized residuals, were examined. In addition, a multivariable logistic regression analysis was employed to assess the association between the preoperative PLT/WBC ratio and postoperative PE. Moreover, restricted cubic splines (RCS) were used to model the dose-response relationship between preoperative PLT/WBC ratio and postoperative PE.

To evaluate the linking role of the PLT/WBC ratio in the association between preoperative chronic steroid use and postoperative PE, we estimated the average causal mediation effect (ACME), average direct effect (ADE), total effect, and proportion of mediation using the ‘mediation’ package in R [38]. A logistic regression model was specified for the binary outcome (postoperative PE) and a linear regression model for the continuous linking factor (PLT/WBC ratio). The proportion of mediation was quantified as the ratio of the ACME to the total effect, expressed as a percentage [39]. Estimates and 95% confidence intervals were calculated using the nonparametric bootstrap method with 1,000 resamples.

To address potential bias from missing data, we conducted a sensitivity analysis using multiple imputation by chained equations [40]. Given that major covariates had substantial missingness (up to 46%), we generated 50 imputed datasets [41]. Subsequently, the primary mediation analysis was replicated on each of these imputed datasets. Rubin’s rules were employed to obtain pooled estimates.

Statistical analyses were conducted utilizing EmpowerStats (X&Y Solutions, Inc., Boston, MA) and R software (version 4.2.0). All statistical tests were two-tailed, with a *P* value of less than 0.05 deemed indicative of statistical significance. To account for multiple comparisons across the primary analyses, the Benjamini-Hochberg false discovery rate procedure was applied to *P* values from the multivariable adjusted models and the mediation analysis. A Benjamini-Hochberg adjusted *P* value < 0.05 was considered statistically significant.

## Results

### Baseline characteristics

A total of 15,274 adult patients who underwent tumor craniotomy were included in the study, among whom 2,319 (15.18%) had a history of preoperative chronic steroid use. The baseline characteristics of the study population are summarized in Table 1. Females accounted for 51.69% of the cohort, and the majority of patients were aged between 41 and 80 years (80.95%). Significant differences in demographic and clinical characteristics were observed between the two groups. Compared to non-steroid users, patients with chronic steroid use had a significantly higher proportion of comorbidities, including diabetes (13.24% vs. 11.65%, *P* = 0.029), disseminated cancer (38.64% vs. 21.24%, *P* < 0.001), and bleeding disorders (3.58% vs. 1.98%, *P* < 0.001). Conversely, the chronic steroid group had a lower proportion of smokers (18.46% vs. 20.48%, *P* = 0.025) and emergency cases (6.17% vs. 7.94%, *P* = 0.003). Furthermore, chronic steroid users were more frequently classified as dependent in terms of functional health status (7.02% vs. 4.04%, *P* < 0.001) and had higher ASA classifications (82.90% vs. 73.94%, *P* < 0.001). In terms of laboratory values, the chronic steroid group had elevated BUN levels, but lower serum sodium and ALB levels compared to the non-steroid group (all *P* < 0.001). Notably, regarding the hypothesized linking factor, the median PLT/WBC ratio for the overall cohort was 26.76 (IQR: 19.28–36.03), with a right-skewed distribution (S1 Fig). Patients with chronic steroid use exhibited a significantly lower median PLT/WBC ratio compared to non-steroid users (22.31 vs. 27.62, *P* < 0.001).

**Table 1:** Baseline characteristics of study participants stratified by preoperative chronic steroid use.

Variables	Overall(n = 15274)	Preoperative chronic steroid use		
		Non-steroid(n = 12955)	Chronic steroid(n = 2319)	*P* value
Age range, n (%)				<0.001
18-40 years	2397 (15.69%)	2111 (16.29%)	286 (12.33%)	
41-60 years	6319 (41.37%)	5336 (41.19%)	983 (42.39%)	
61-80 years	6046 (39.58%)	5069 (39.13%)	977 (42.13%)	
≥ 81 years	512 (3.35%)	439 (3.39%)	73 (3.15%)	
Sex, n (%)				0.833
Female	7895 (51.69%)	6701 (51.73%)	1194 (51.49%)	
Male	7379 (48.31%)	6254 (48.27%)	1125 (48.51%)	
Race, n (%)				0.139
White	10783 (70.60%)	9159 (70.70%)	1624 (70.03%)	
Black	1034 (6.77%)	893 (6.89%)	141 (6.08%)	
Other	3457 (22.63%)	2903 (22.41%)	554 (23.89%)	
Smoking status, n (%)				0.025
No	12193 (79.83%)	10302 (79.52%)	1891 (81.54%)	
Yes	3081 (20.17%)	2653 (20.48%)	428 (18.46%)	
BMI, n (%)				0.758
< 25.0 kg/m^2^	4599 (31.56%)	3899 (31.58%)	700 (31.43%)	
25.0-29.9 kg/m^2^	4945 (33.93%)	4201 (34.03%)	744 (33.41%)	
≥ 30.0 kg/m^2^	5028 (34.50%)	4245 (34.39%)	783 (35.16%)	
Diabetes, n (%)				0.029
No	13458 (88.11%)	11446 (88.35%)	2012 (86.76%)	
Yes	1816 (11.89%)	1509 (11.65%)	307 (13.24%)	
Hypertension, n (%)				0.303
No	9407 (61.59%)	8001 (61.76%)	1406 (60.63%)	
Yes	5867 (38.41%)	4954 (38.24%)	913 (39.37%)	
Disseminated cancer, n (%)				<0.001
No	11626 (76.12%)	10203 (78.76%)	1423 (61.36%)	
Yes	3648 (23.88%)	2752 (21.24%)	896 (38.64%)	
Bleeding disorders, n (%)				<0.001
No	14935 (97.78%)	12699 (98.02%)	2236 (96.42%)	
Yes	339 (2.22%)	256 (1.98%)	83 (3.58%)	
Preoperative sepsis, n (%)				0.807
No	14614 (95.68%)	12393 (95.66%)	2221 (95.77%)	
Yes	660 (4.32%)	562 (4.34%)	98 (4.23%)	
Emergency case, n (%)				0.003
No	14103 (92.33%)	11927 (92.06%)	2176 (93.83%)	
Yes	1171 (7.67%)	1028 (7.94%)	143 (6.17%)	
Functional health status, n (%)				<0.001
Independent	14525 (95.51%)	12380 (95.96%)	2145 (92.98%)	
Dependent	683 (4.49%)	521 (4.04%)	162 (7.02%)	
ASA classification, n (%)				<0.001
Class I-II	3736 (24.70%)	3344 (26.06%)	392 (17.10%)	
Class III-V	11387 (75.30%)	9486 (73.94%)	1901 (82.90%)	
BUN, mg/dL, median (Q1-Q3)	16.00(12.00-21.00)	16.00(12.00-20.00)	19.00(14.00-24.00)	<0.001
AST, U/L, median (Q1-Q3)	21.00(16.00-28.00)	21.00(17.00-27.00)	21.00(16.00-29.00)	0.846
Sodium, mmol/L, mean ± SD	138.51 ± 3.25	138.65 ± 3.18	137.73 ± 3.56	<0.001
ALB, g/dL, mean ± SD	3.86 ± 0.57	3.88 ± 0.57	3.77 ± 0.57	<0.001
PLT/WBC ratio, median (Q1-Q3)	26.76(19.28-36.03)	27.62(20.09-36.81)	22.31(16.10-30.46)	<0.001

In the study involving 15,274 participants, the proportion of missing data for the variables under investigation was 702 (4.60%) for BMI, 66 (0.43%) for functional health status, 151 (0.99%) for ASA classification, 887 (5.81%) for BUN, 7,091 (46.43%) for AST, 308 (3.73%) for sodium, and 7,024 (45.98%) for ALB. ALB, albumin; ASA, American Society of Anesthesiologists; AST, Aspartate Aminotransferase; BMI, body mass index; BUN, blood urea nitrogen; PLT/WBC, platelet-to-white blood cell; SD, standard deviation.

### Rate of 30-day postoperative PE

In our cohort, the overall rate of 30-day postoperative PE was 1.41% (215/15,274). Patients in the chronic steroid group exhibited a significantly higher PE rate compared to the non-steroid group (2.37% [55/2,319] vs. 1.24% [160/12,955], *P* < 0.001).

### Association between preoperative chronic steroid use and postoperative PE

Table 2 illustrates the relationship between preoperative chronic steroid use and postoperative PE. In the crude model, preoperative chronic steroid use was significantly associated with higher odds of PE (OR = 1.94, 95% CI: 1.43, 2.65; *P* < 0.001). This association remained robust after adjusting for demographic factors in Model 1 (OR = 1.93, 95% CI: 1.41, 2.63; *P* < 0.001). Further adjustment for additional covariates (Model 2) continued to show a significant association between preoperative chronic steroid use and PE (OR = 1.90, 95% CI: 1.24, 2.90; *P* = 0.003). Due to missing data for certain covariates (notably AST and ALB), the number of complete cases available for Model 2 was 7,360, comprising 115 PE events.

**Table 2:** The association between preoperative chronic steroid use and postoperative PE in adult tumor craniotomy.

Exposure	Crude model		Model 1		Model 2	
	OR (95% CI)	*P* value	OR (95% CI)	*P* value	OR (95% CI)	*P* value
Non-steroid	1.00 (Reference)		1.00 (Reference)		1.00 (Reference)	
Chronic steroid	1.94 (1.43, 2.65)	< 0.001	1.93 (1.41, 2.63)	< 0.001	1.90 (1.24, 2.90)	0.003

Model 1: adjusted for age range, sex and race (n = 15,274; 215 PE events); Model 2: adjusted for age range, sex, race, smoking status, body mass index, diabetes, hypertension, disseminated cancer, bleeding disorders, preoperative sepsis, emergency case, functional health status, American Society of Anesthesiologists classification, blood urea nitrogen, aspartate aminotransferase, sodium, and albumin (n = 7,360; 115 PE events). CI, confidence interval; OR, odds ratio; PE, pulmonary embolism.

Subgroup analyses were performed to assess the consistency of the association between preoperative chronic steroid use and PE across diverse demographic and clinical characteristics (S1 Table). The results demonstrated that the association was largely consistent across the majority of subgroups. Notably, no significant interactions were observed between chronic steroid use and any of the stratified variables (all *P* for interaction > 0.05), thereby reinforcing the robustness of the observed association.

### Association between preoperative chronic steroid use and PLT/WBC ratio

Table 3 presents the results of the linear regression analysis examining the relationship between preoperative chronic steroid use and PLT/WBC ratio. In the multivariable adjusted model (Model 2), preoperative chronic steroid use was significantly and negatively associated with PLT/WBC ratio (β = −3.02, 95% CI: −3.82, −2.22; *P* < 0.001). The Normal Q-Q plot of standardized residuals from the multivariable linear regression model is presented in S2 Fig.

**Table 3:** The association between preoperative chronic steroid use and PLT/WBC ratio in adult tumor craniotomy.

Exposure	Crude model		Model 1		Model 2	
	β (95% CI)	*P* value	β (95% CI)	*P* value	β (95% CI)	*P* value
Non-steroid	0.00 (Reference)		0.00 (Reference)		0.00 (Reference)	
Chronic steroid	−5.29 (−6.52, −4.07)	< 0.001	−5.17 (−6.39, −3.95)	< 0.001	−3.02 (−3.82, −2.22)	< 0.001

Model 1: adjusted for age range, sex and race (n = 15,274; 215 PE events); Model 2: adjusted for age range, sex, race, smoking status, body mass index, diabetes, hypertension, disseminated cancer, bleeding disorders, preoperative sepsis, emergency case, functional health status, American Society of Anesthesiologists classification, blood urea nitrogen, aspartate aminotransferase, sodium, and albumin (n = 7,360; 115 PE events). CI, confidence interval; PLT/WBC, platelet-to-white blood cell; PE, pulmonary embolism.

### Association between PLT/WBC ratio and postoperative PE

We further evaluated the relationship between PLT/WBC ratio and the odds of postoperative PE, as summarized in Table 4. In the continuous analysis, each one-unit increase in PLT/WBC ratio was associated with a 3% reduction in the odds of postoperative PE (OR = 0.97, 95% CI: 0.95, 0.99; *P* = 0.001). When PLT/WBC ratio was categorized into tertiles, a lower ratio was associated with higher odds of PE. In the multivariable adjusted model (Model 2), patients in the lowest tertile (Tertile 1) had significantly higher odds of PE compared to those in the highest tertile (Tertile 3) (OR = 2.23, 95% CI: 1.31, 3.78; *P* = 0.003). Furthermore, a significant trend towards higher ORs with decreasing PLT/WBC ratio was observed across the tertiles (*P* for trend = 0.002). This inverse association was further examined using RCS (S3 Fig), which demonstrated a linear dose-response relationship (*P* for overall = 0.009; *P* for nonlinear = 0.798).

**Table 4:** The association between preoperative PLT/WBC ratio and postoperative PE in adult tumor craniotomy.

Exposure	Crude model		Model 1		Model 2	
	OR (95% CI)	*P* value	OR (95% CI)	*P* value	OR (95% CI)	*P* value
PLT/WBC ratio	0.97 (0.96, 0.98)	< 0.001	0.98 (0.96, 0.99)	< 0.001	0.97 (0.95, 0.99)	0.001
PLT/WBC ratio tertile						
Tertile 3 (32.41–2810.00)	1.00 (Reference)		1.00 (Reference)		1.00 (Reference)	
Tertile 2 (21.75–32.41)	1.14 (0.78, 1.68)	0.493	1.11 (0.75, 1.63)	0.612	1.35 (0.77, 2.37)	0.290
Tertile 1 (0.66–21.75)	2.27 (1.62, 3.19)	< 0.001	2.12 (1.50, 3.00)	< 0.001	2.23 (1.31, 3.78)	0.003
*P* for trend	< 0.001		< 0.001		0.002	

Model 1: adjusted for age range, sex and race (n = 15,274; 215 PE events); Model 2: adjusted for age range, sex, race, smoking status, body mass index, diabetes, hypertension, disseminated cancer, bleeding disorders, preoperative sepsis, emergency case, functional health status, American Society of Anesthesiologists classification, blood urea nitrogen, aspartate aminotransferase, sodium, and albumin (n = 7,360; 115 PE events). CI, confidence interval; OR, odds ratio; PE, pulmonary embolism; PLT/WBC, platelet-to-white blood cell.

### Linking role of PLT/WBC ratio

**Fig 2:**
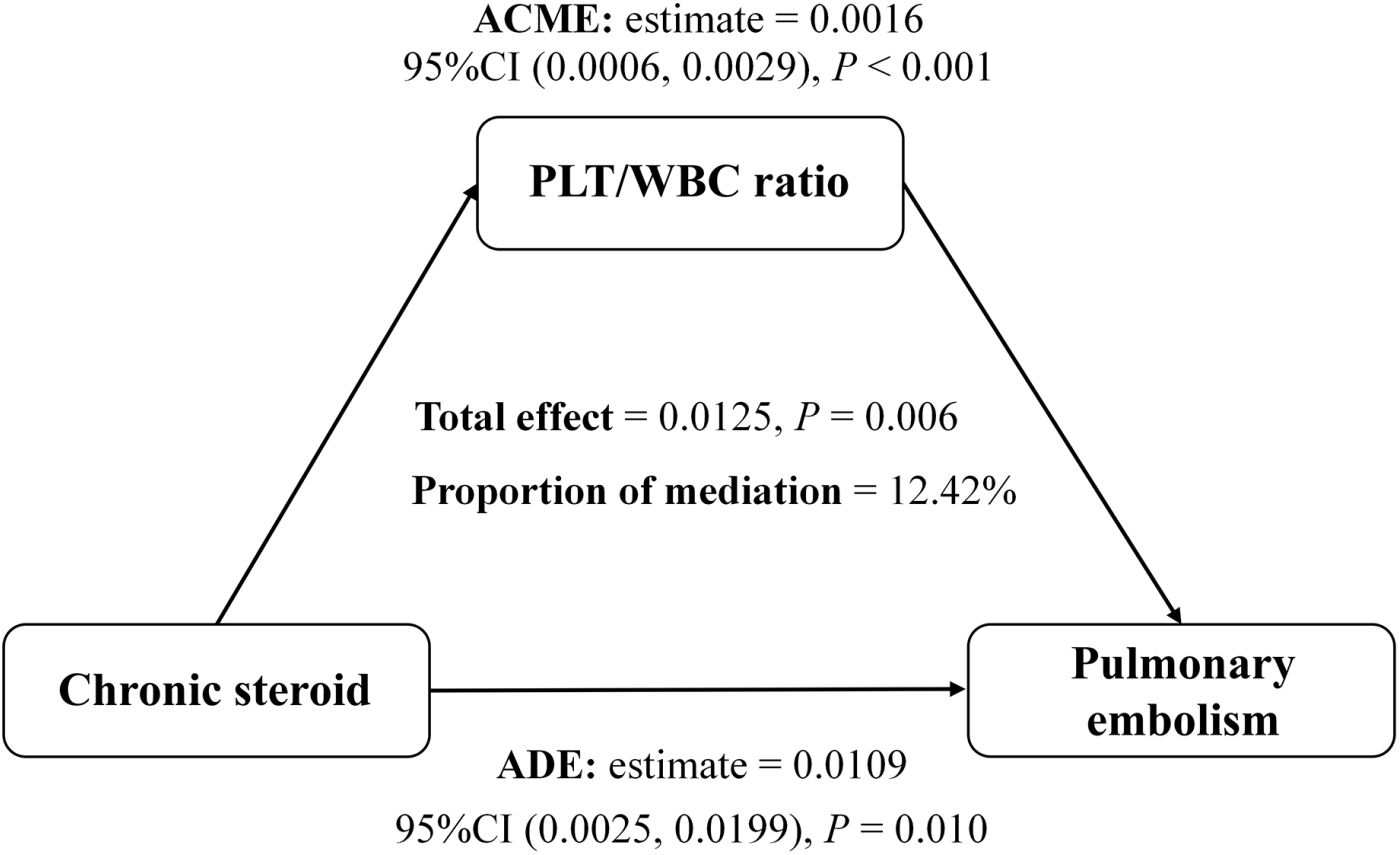
Mediation analysis of PLT/WBC ratio on the association between preoperative chronic steroid use and postoperative pulmonary embolism. Adjusted for age range, sex, race, smoking status, body mass index, diabetes, hypertension, disseminated cancer, bleeding disorders, preoperative sepsis, emergency case, functional health status, American Society of Anesthesiologists classification, blood urea nitrogen, aspartate aminotransferase, sodium, and albumin. Estimates and 95% confidence intervals were calculated using the nonparametric bootstrap method with 1,000 resamples. ACME, average causal mediation effect; ADE, average direct effect; PLT/WBC, platelet-to-white blood cell.

Building upon the significant associations observed in the preceding sections, a mediation analysis was performed to elucidate the role of the PLT/WBC ratio in the relationship between preoperative chronic steroid use and postoperative PE. As depicted in Fig 2, the mediation analysis revealed a significant ADE of preoperative chronic steroid use on postoperative PE (estimate = 0.0109, 95% CI: 0.0025, 0.0199; *P* = 0.010). The ACME through PLT/WBC ratio was also statistically significant (estimate = 0.0016, 95% CI: 0.0006, 0.0029; *P* < 0.001), with a total effect estimate of 0.0125 (95% CI: 0.0040, 0.0215; *P* = 0.006). Consequently, approximately 12.42% of the total effect of preoperative chronic steroid use on postoperative PE was mediated by PLT/WBC ratio (*P* = 0.006). The findings remained consistent across models, with the proportion of mediation varying from 17.94% in the crude model to 15.98% in the model adjusting for demographics (Table 5).

**Table 5:** Mediation analysis of the role of PLT/WBC ratio in the association between preoperative chronic steroid use and postoperative PE.

Analysis	Crude model	Model 1	Model 2
	Estimate (95% CI) *P* value	Estimate (95% CI) *P* value	Estimate (95% CI) *P* value
ADE	0.0103 (0.0037, 0.0177)0.006	0.0102 (0.0036, 0.0175)0.004	0.0109 (0.0025, 0.0199)0.010
ACME	0.0022 (0.0010, 0.0045)<0.001	0.0019 (0.0007, 0.0039)<0.001	0.0016 (0.0006, 0.0029)<0.001
Total effect	0.0125 (0.0055, 0.0210)<0.001	0.0121 (0.0053, 0.0203)<0.001	0.0125 (0.0040, 0.0215)0.006
Proportion ofmediation (%)	17.94 (8.11, 41.60)<0.001	15.98 (6.38, 39.50)<0.001	12.42 (4.88, 38.32)0.006

Model 1: adjusted for age range, sex and race (n = 15,274; 215 PE events); Model 2: adjusted for age range, sex, race, smoking status, body mass index, diabetes, hypertension, disseminated cancer, bleeding disorders, preoperative sepsis, emergency case, functional health status, American Society of Anesthesiologists classification, blood urea nitrogen, aspartate aminotransferase, sodium, and albumin (n = 7,360; 115 PE events). Estimates and 95% confidence intervals were calculated using the nonparametric bootstrap method with 1,000 resamples. ACME, average causal mediation effect; ADE, average direct effect; PE, pulmonary embolism; PLT/WBC, platelet-to-white blood cell ratio.

To address the concern of multiple comparisons, the Benjamini-Hochberg procedure was applied to the *P* values derived from the primary multivariable adjusted models and mediation analysis. All statistically significant findings from these primary analyses remained significant after adjustment (all Benjamini-Hochberg adjusted *P* < 0.05).

### Sensitivity analysis

The robustness of our primary findings was corroborated through sensitivity analyses. First, to address the potential bias arising from missing data, mediation analyses were repeated using 50 multiply imputed datasets (S2 Table). Consistent with the main results, the linking role of PLT/WBC ratio remained statistically significant across all datasets (all *P* < 0.05), with a pooled proportion of mediation of 8.95% (*P* = 0.002). Furthermore, to capture PLT/WBC ratio with closer proximity to the surgical procedure, we restricted the analysis to participants (n = 4,770) with available platelet and WBC data within the two days preceding surgery (S3 Table). The results demonstrated that the finding was consistent, with a proportion of mediation of 9.94% (*P* = 0.040). Third, to assess the potential implications of excluding patients with no available platelet or WBC measurements within the 10 days preceding surgery, we compared the baseline characteristics between the included (n = 15,274) and excluded (n = 3,368) patients. The results are presented in S4 Table. Several statistically significant differences were observed between the two groups, whereas the rates of preoperative chronic steroid use and postoperative PE were broadly comparable across the two populations. Fourth, we conducted an additional sensitivity analysis using a more parsimonious adjustment model by excluding AST and ALB (n = 13,564; 190 PE events). The mediation analysis demonstrated that PLT/WBC ratio continued to significantly explain part of the association between preoperative chronic steroid use and postoperative PE, with a proportion of mediation of 13.55% (*P* < 0.001) (S5 Table). Fifth, to evaluate whether the high proportion of missing data for AST (46.43%) and ALB (45.98%) influenced the primary associations, we compared baseline characteristics (S6 Table) and the primary associations between participants with complete ALB and AST data (n = 7,694) and those with missing values (n = 7,580). Among participants with complete ALB and AST data, the association between preoperative chronic steroid use and postoperative PE remained significant (OR = 2.01, 95% CI: 1.32, 3.07) (S7 Table), and the linking role of PLT/WBC ratio was also significant (proportion of mediation: 13.58%, *P* = 0.002) (S8 Table). Among participants with missing ALB or AST, the associations were directionally consistent but did not reach statistical significance (S9 and S10 Tables), likely due to limited statistical power given fewer PE events in this subgroup. Finally, to evaluate whether the observed linking role was specifically attributable to the PLT/WBC ratio rather than its individual components, separate mediation analyses were performed using platelet count and WBC count as linking factors. Neither platelet count (proportion of mediation: 5.06%, *P* = 0.090; S11 Table) nor WBC count (proportion of mediation: 2.10%, *P* = 0.100; S12 Table) individually demonstrated a statistically significant linking role, supporting that the PLT/WBC ratio captures integrative information beyond its components.

## Discussion

In this retrospective cohort study leveraging the ACS-NSQIP database, we examined the relationship between preoperative chronic steroid exposure and postoperative PE among 15,274 adult patients undergoing craniotomy for tumors. Our findings demonstrated that preoperative chronic steroid use was significantly associated with elevated odds of PE (OR = 1.90, 95% CI: 1.24, 2.90). Additionally, we observed that steroid use was associated with lower preoperative PLT/WBC ratio, and a lower PLT/WBC ratio was linked to higher odds of postoperative PE. Building on these associations, mediation analysis revealed that the PLT/WBC ratio partially linked the relationship between preoperative steroid use and postoperative PE, accounting for approximately 12.42% of the total effect. These findings were further supported by sensitivity analyses, providing initial evidence that the PLT/WBC ratio may serve as a potential linking factor between preoperative chronic steroid use and 30-day postoperative PE in this population.

The positive association between preoperative chronic steroid use and the occurrence of postoperative PE observed in our study aligns with and reinforces existing evidence within the neurosurgical literature [17,19,36]. Most notably, our finding (OR = 1.90) is consistent with a previous retrospective cohort study focused specifically on patients undergoing craniotomy for tumor resection, which reported chronic steroid use as a significant risk factor for PE with a comparable effect size (OR = 1.76) [18]. Beyond this specific population, this relationship has also been documented in broader cohorts of neurosurgical patients, where database analyses have consistently demonstrated increased odds of PE among chronic steroid users, with reported ORs ranging from approximately 1.47 to 1.63 [17,19]. Furthermore, studies examining the composite outcome of VTE in brain tumor patients have similarly corroborated the elevated odds associated with chronic steroid use [36]. Collectively, these investigations, in conjunction with our current results, support the link between preoperative chronic steroid use and postoperative PE in patients undergoing craniotomy. However, while this clinical association is well-documented, the specific intermediate links explaining this relationship remain to be fully elucidated, prompting further investigation into the potential linking role of the PLT/WBC ratio.

Understanding the impact of preoperative chronic steroid use on the hematological profile is essential for interpreting the linking role of the PLT/WBC ratio. Our analysis revealed a negative association between steroid use and this ratio, a finding that is clinically consistent with the differential effects of glucocorticoids on blood cell lineages. It is widely documented that systemic corticosteroid administration is associated with significant leukocytosis [22]. Conversely, the influence of steroids on platelets is more nuanced. They are, for example, therapeutically used to increase platelet counts in conditions like immune thrombocytopenia [24]. The significant reduction in the PLT/WBC ratio observed among steroid users in our study suggests that the notable leukocytosis characteristic of this group likely disproportionately outweighs any concurrent changes in platelet counts, thereby explaining the downward shift in the ratio.

Linking this hematological shift to clinical outcomes, our multivariable analysis demonstrated that a lower PLT/WBC ratio was associated with increased odds of postoperative PE. Although data specifically linking this ratio to VTE in neurosurgical patients undergoing tumor craniotomy are limited, our findings parallel established associations in other thrombo-inflammatory vascular diseases. For example, a cohort study involving over 27,000 participants observed a significant inverse relationship between the PLT/WBC ratio and the risk of fatal stroke [27]. Similarly, in patients with acute ischemic stroke, including those receiving intravenous thrombolysis, a lower PLT/WBC ratio has been independently linked to unfavorable functional outcomes and increased neurological severity [28,29]. Across these thrombo-inflammatory conditions, a decreased ratio may reflect a systemic imbalance characterized by elevated circulating leukocyte relative to platelet counts, a condition conducive to hypercoagulability. By extending these observations to our specific cohort, our study suggests that the PLT/WBC ratio may function as a biologically plausible hematological index associated with the odds of PE, serving as an intermediate link in the association between chronic steroid use and postoperative PE.

The mediation analysis conducted in this study further indicated that the PLT/WBC ratio may partially explain the relationship between preoperative chronic steroid use and the odds of 30-day postoperative PE. While the observational design of our study precludes definitive causal inferences, existing literature regarding the inflammation-coagulation axis offers several biologically plausible mechanisms that may underlie these observed associations. First, a lower PLT/WBC ratio, predominantly reflected by the pronounced leukocytosis relative to platelet counts observed in the chronic steroid use group, implies an expanded reservoir of circulating leukocytes. Within this intravascular environment, the increased abundance of neutrophils amplifies the pool of effector cells available for activation, thereby increasing the propensity for the extrusion of neutrophil extracellular traps (NETs) [42]. These NETs may not only physically entrap platelets but also directly activate the intrinsic coagulation pathway (e.g., Factor XII) and degrade natural anticoagulants like antithrombin III, thereby promoting venous thrombogenesis [43–45]. Second, the altered hematological composition suggested by this ratio may favor the formation of leukocyte-platelet aggregates [28]. Facilitated by adhesion molecules such as P-selectin, the interaction between platelets and the expanded leukocyte pool can enhance leukocyte recruitment to the vascular endothelium [46]. These aggregates can provide a procoagulant surface that facilitates the activation of the coagulation cascade, potentially creating a feed-forward loop that exacerbates endothelial injury and localized hypercoagulability [47,48]. Third, the disruption of hemostatic equilibrium captured by a low PLT/WBC ratio may be associated with a systemic release of pro-thrombotic factors [49]. This hematological shift may correlate with an increased systemic burden of tissue factor-bearing microparticles released from the expanded leukocyte population, which collectively shift the hemostatic balance towards a hypercoagulable state [50]. Consequently, the PLT/WBC ratio may reflect these complex thrombo-inflammatory interactions, serving as a readily calculable hematological marker associated with the pathways linking preoperative chronic steroid use to the odds of postoperative PE. Importantly, this finding should not be interpreted as evidence that the PLT/WBC ratio is ready to directly guide anticoagulation decisions. Rather, the PLT/WBC ratio is best conceptualized as a low-cost, routinely available risk marker that may help explore steroid-exposed tumor craniotomy patients who warrant more systematic attention to perioperative VTE prevention planning.

From a clinical perspective, our findings may have implications for the perioperative management of adult tumor craniotomy patients with chronic steroid exposure. Given the association between chronic steroid use and elevated PE odds observed in this and prior studies, the recognition of preoperative chronic steroid exposure in this population could reasonably prompt a structured perioperative thromboembolism risk review. Based on established perioperative VTE prevention principles [51,52], such a review may include systematic documentation of mobility limitations, early initiation of mechanical prophylaxis (e.g., intermittent pneumatic compression devices), early postoperative mobilization when neurologically feasible, and the establishment of a clear plan for the timing of pharmacological thromboprophylaxis once adequate neurosurgical hemostasis has been achieved. The PLT/WBC ratio, as a component of routinely collected preoperative laboratory data, could potentially supplement such risk stratification efforts by flagging patients with hematological profiles suggestive of heightened PE risk. Nevertheless, the integration of this marker into clinical decision-making algorithms awaits validation through prospective studies specifically designed to evaluate its clinical utility in this setting.

While our findings offer valuable clinical insights, several methodological constraints inherent to our study design should be acknowledged. First, the retrospective nature of this ACS-NSQIP database analysis inherently restricts our ability to establish definitive causal relationships between preoperative chronic steroid use, the PLT/WBC ratio, and the odds of postoperative PE, rendering our findings strictly observational. Furthermore, the possibility of residual confounding cannot be entirely eliminated, as the registry lacks clinical details concerning the precise indications for steroid therapy (e.g., dexamethasone for peritumoral edema vs. corticosteroids for chronic conditions such as chronic obstructive pulmonary disease or rheumatologic disease), cumulative drug dosages, specific durations of treatment, and granular tumor biology profiles including histological subtype and malignancy grade. These unmeasured variables may be independently associated with both the likelihood of chronic steroid exposure and the odds of postoperative PE, representing a residual source of confounding that cannot be fully addressed with the available data. Moreover, the inability to distinguish between different clinical indications for steroid use may introduce heterogeneity within the exposure group, potentially attenuating or obscuring true effect estimates. These limitations are inherent to the use of secondary registry data and should be considered when interpreting the observed associations. Future studies with detailed medication records and comprehensive tumor pathology data are warranted to disentangle these potentially distinct clinical scenarios. Second, a critical limitation pertains to the absence of perioperative VTE prophylaxis data within the ACS-NSQIP database. Specifically, information regarding the timing of pharmacological anticoagulation, the use of mechanical prophylaxis devices, concerns related to postoperative hemorrhage that may have influenced prophylaxis decisions, and detailed postoperative mobility data were not available for analysis. Without these data, we cannot determine whether the observed association between chronic steroid use and PE was modified by differential application of prophylactic measures, nor can our findings be directly translated into specific treatment recommendations. Future studies with detailed prophylaxis and mobilization data are needed to clarify these relationships. Third, evaluating the linking role of the PLT/WBC ratio using baseline preoperative data introduces an inherent risk of temporal ambiguity between the exposure and the presumed linking factor. To mitigate this, we strictly applied the ACS-NSQIP criteria for chronic steroid use (defined as administration for >10 days within the 30 days preceding surgery) while restricting the measurement of the PLT/WBC ratio to within 10 days prior to surgery. This design ensures that the period of chronic steroid exposure preceded or overlapped with the linking factor measurement window. However, the 10-day preoperative window may not precisely reflect the hematological state at the time of surgery, as steroid-induced leukocytosis can fluctuate substantially over this period. A sensitivity analysis restricting the PLT/WBC ratio measurement to within 2 days prior to surgery demonstrated a consistent linking role (proportion of mediation: 9.94%, *P* = 0.040). Nevertheless, this restricted analysis included only 4,770 of 15,274 patients (31.23%), indicating that for the remaining majority of the primary cohort, the linking factor measurement had limited temporal precision relative to the operative date. Despite these design measures, the temporal sequence between chronic steroid exposure and the PLT/WBC ratio cannot be definitively established within the constraints of observational data, and our findings should be interpreted accordingly. Fourth, substantial missing data for certain covariates, most notably AST (46.43%) and ALB (45.98%), may reduce the precision of effect estimates and could introduce bias. To address this, multiple imputation with 50 datasets and a parsimonious sensitivity analysis excluding the two covariates were both performed. The linking role of the PLT/WBC ratio remained statistically significant and directionally consistent across these approaches. Future studies utilizing datasets with more complete covariate documentation are warranted to provide more precise effect estimates. Fifth, regarding the assessment of our outcome, the ACS-NSQIP defines postoperative PE based on a “new diagnosis” within 30 days after the principal operation. Because routine preoperative pulmonary imaging is not typically mandated, we cannot definitively exclude the possibility that some cases identified as postoperative PE were, in fact, preexisting occult PEs that only became symptomatic or were first detected postoperatively. This inherent diagnostic detection bias may lead to a degree of outcome misclassification. Furthermore, as we utilized a secondary dataset, the days-to-event variable (DPULEMBOL) was unavailable, preventing us from performing temporal consistency checks on the occurrence of the outcome. Sixth, the potential for detection bias must be acknowledged. As demonstrated in our baseline characteristics (Table 1), patients exposed to chronic steroids often presented with a heavier comorbidity burden, such as higher rates of disseminated cancer and higher ASA classifications. Consequently, these patients may be hospitalized longer and subjected to closer clinical monitoring and more frequent imaging. Because PE ascertainment in the ACS-NSQIP relies on clinically prompted imaging leading to a new diagnosis, rather than a systematic screening protocol applied uniformly to all patients, the higher rate of PE observed in the steroid group might partially reflect an increased detection of incidental or asymptomatic PEs rather than a purely true increase in incidence. Seventh, because the data were obtained from the supplementary material of the original article rather than directly accessed from the ACS-NSQIP database, the variable definitions in the extracted dataset may not perfectly align with the ACS-NSQIP user guide specifications relied upon in the current study. Specifically, the coding of preoperative chronic steroid use, postoperative PE, and other covariates was determined by the original authors’ extraction conventions, which may differ subtly from our stated definitions. This introduces a potential risk of exposure, outcome, and covariate misclassification that should be acknowledged when interpreting our findings. Finally, because the database predominantly captures information from participating centers within the United States, the applicability of our findings to other demographic groups or healthcare systems may be constrained. Additionally, as our analytical cohort was restricted to patients with available preoperative platelet and WBC measurements within 10 days before surgery, the findings may not be directly generalizable to patients lacking such laboratory data. As shown in S4 Table, the excluded patients differed from the included cohort across several baseline characteristics, including a higher proportion of younger patients and those with fewer comorbidities, suggesting that the excluded individuals may represent a clinically distinct subpopulation. Caution is therefore warranted when extrapolating these findings beyond the study cohort. Future investigations involving diverse international cohorts are warranted to corroborate these observed associations across different clinical settings.

## Conclusion

In this retrospective cohort of 15,274 adults undergoing tumor craniotomy drawn from the ACS-NSQIP database, our findings further support that preoperative chronic steroid use was associated with elevated odds of postoperative PE. More importantly, our analysis revealed that preoperative PLT/WBC ratio may act as a partial linking factor in this relationship. Future investigations are necessary to substantiate these findings.

## Supporting information

S1 Fig Histogram of preoperative PLT/WBC ratio. PLT/WBC, platelet-to-white blood cell.
(TIF)

S2 Fig Normal Q-Q plot of standardized residuals from the multivariable linear regression model assessing the association between preoperative chronic steroid use and platelet-to-white blood cell ratio. (TIF)

S3 Fig Restricted cubic spline analysis of the dose-response relationship between preoperative PLT/WBC ratio and postoperative PE. The reference point is the median PLT/WBC ratio. Adjusted for age range, sex, race, smoking status, body mass index, diabetes, hypertension, disseminated cancer, bleeding disorders, preoperative sepsis, emergency case, functional health status, American Society of Anesthesiologists classification, blood urea nitrogen, aspartate aminotransferase, sodium, and albumin. PE, pulmonary embolism; PLT/WBC, platelet-to-white blood cell.
(TIF)

S1 Table Subgroup analysis: stratified associations between preoperative chronic steroid use and postoperative PE in adult tumor craniotomy. (DOCX)

S2 Table Sensitivity analysis: mediation analysis of the role of PLT/WBC ratio in the association between preoperative chronic steroid use and postoperative PE across 50 multiple imputation datasets. (DOCX)

S3 Table Sensitivity analysis: mediation analysis of the role of PLT/WBC ratio in the association between preoperative chronic steroid use and postoperative PE in participants with available PLT and WBC data within 2 days prior to surgery. (DOCX)

S4 Table Sensitivity analysis: comparison of characteristics between the included and excluded patients. (DOCX)

S5 Table Sensitivity analysis: mediation analysis of the role of PLT/WBC ratio in the association between preoperative chronic steroid use and postoperative PE in parsimonious model. (DOCX)

S6 Table Sensitivity analysis: comparison of characteristics between participants with complete ALB and AST data and those with missing ALB or AST data. (DOCX)

S7 Table Sensitivity analysis: the association between preoperative chronic steroid use and postoperative PE among participants with complete ALB and AST data. (DOCX)

S8 Table Sensitivity analysis: mediation analysis of the role of PLT/WBC ratio in the association between preoperative chronic steroid use and postoperative PE among participants with complete ALB and AST data. (DOCX)

S9 Table Sensitivity analysis: the association between preoperative chronic steroid use and postoperative PE among participants with missing ALB or AST data. (DOCX)

S10 Table Sensitivity analysis: mediation analysis of the role of PLT/WBC ratio in the association between preoperative chronic steroid use and postoperative PE among participants with missing ALB or AST data. (DOCX)

S11 Table Sensitivity analysis: mediation analysis of the role of PLT count in the association between preoperative chronic steroid use and postoperative PE. (DOCX)

S12 Table Sensitivity analysis: mediation analysis of the role of WBC count in the association between preoperative chronic steroid use and postoperative PE. (DOCX)
