# Supplementary material for: Association between preoperative chronic steroid use and pulmonary embolism in adult tumor craniotomy: Linking role of platelet-to-white blood cell ratio
Source: PLoS One. 2026 Aug 4;21(8):e0355416. doi: 10.1371/journal.pone.0355416 (PMC13436738; doi:10.1371/journal.pone.0355416)
Supplement: Supplementary file 1 [file pone.0355416.s004.docx]

**S1 Table. Subgroup analysis: stratified associations between preoperative chronic steroid use and postoperative PE in adult tumor craniotomy.**

| **Stratified variable** | | **Events (%)** | OR (95%CI) P value | P for  interaction |
| --- | --- | --- | --- | --- |
| Age range | 18-40 years | 28 (1.17) | 2.88 (0.94, 8.83) 0.064 | 0.467 |
|  | 41-60 years | 76 (1.20) | 1.09 (0.48, 2.44) 0.838 |  |
|  | 61-80 years | 104 (1.72) | 2.17 (1.19, 3.96) 0.012 |  |
|  | ≥ 81 years | 7 (1.37) | 0.61 (0.02, 16.88) 0.773 |  |
| Sex | Female | 95 (1.20) | 1.78 (0.92, 3.44) 0.087 | 0.665 |
|  | Male | 120 (1.63) | 1.88 (1.07, 3.31) 0.029 |  |
| Race | White | 156 (1.45) | 1.49 (0.90, 2.47) 0.121 | 0.073 |
|  | Black | 17 (1.64) | 0.57 (0.06, 5.15) 0.613 |  |
|  | Other | 42 (1.21) | 6.12 (2.18, 17.16) < 0.001 |  |
| Smoking status | No | 182 (1.49) | 1.89 (1.19, 3.00) 0.007 | 0.581 |
|  | Yes | 33 (1.07) | 1.32 (0.41, 4.23) 0.644 |  |
| BMI | < 25.0 kg/m^2^ | 47 (1.02) | 2.43 (1.06, 5.62) 0.037 | 0.909 |
|  | 25.0-29.9 kg/m^2^ | 61 (1.23) | 1.47 (0.67, 3.22) 0.340 |  |
|  | ≥ 30.0 kg/m^2^ | 96 (1.91) | 1.63 (0.85, 3.13) 0.143 |  |
| Diabetes | No | 181 (1.34) | 1.57 (0.99, 2.51) 0.056 | 0.163 |
|  | Yes | 34 (1.87) | 4.78 (1.37, 16.66) 0.014 |  |
| Hypertension | No | 119 (1.27) | 1.95 (1.12, 3.39) 0.019 | 0.426 |
|  | Yes | 96 (1.64) | 1.49 (0.75, 2.96) 0.251 |  |
| Disseminated cancer | No | 168 (1.45) | 1.43 (0.82, 2.49) 0.210 | 0.095 |
|  | Yes | 47 (1.29) | 2.33 (1.11, 4.87) 0.025 |  |
| Bleeding disorders | No | 210 (1.41) | 1.73 (1.13, 2.67) 0.012 | NA |
|  | Yes | 5 (1.47) | NA^*^ |  |
| Preoperative sepsis | No | 199 (1.36) | 1.74 (1.11, 2.72) 0.015 | 0.934 |
|  | Yes | 16 (2.42) | 4.74 (0.82, 27.55) 0.083 |  |
| Emergency case | No | 198 (1.40) | 1.80 (1.16, 2.78) 0.009 | 0.684 |
|  | Yes | 17 (1.45) | 0.91 (0.07, 12.01) 0.941 |  |
| Functional health status | Independent | 197 (1.36) | 1.68 (1.06, 2.67) 0.027 | 0.371 |
|  | Dependent | 15 (2.20) | 2.67 (0.68, 10.47) 0.160 |  |
| ASA classification | Class I-II | 34 (0.91) | 3.90 (0.94, 16.08) 0.060 | 0.748 |
|  | Class III-V | 176 (1.55) | 1.64 (1.05, 2.57) 0.031 |  |
| BUN | Low | 52 (1.11) | 1.55 (0.60, 3.95) 0.364 | 0.728 |
|  | Middle | 61 (1.36) | 2.58 (1.13, 5.86) 0.024 |  |
|  | High | 95 (1.82) | 1.48 (0.80, 2.73) 0.212 |  |
| AST | Low | 33 (1.32) | 2.05 (0.89, 4.75) 0.093 | 0.854 |
|  | Middle | 47 (1.64) | 1.56 (0.75, 3.22) 0.233 |  |
|  | High | 47 (1.67) | 1.87 (0.91, 3.83) 0.089 |  |
| Sodium | Low | 57 (1.63) | 2.40 (1.16, 4.97) 0.018 | 0.079 |
|  | Middle | 82 (1.44) | 2.11 (1.05, 4.25) 0.036 |  |
|  | High | 72 (1.25) | 0.83 (0.31, 2.20) 0.708 |  |
| ALB | Low | 56 (2.16) | 1.56 (0.82, 2.96) 0.174 | 0.533 |
|  | Middle | 42 (1.78) | 2.32 (1.12, 4.80) 0.023 |  |
|  | High | 35 (1.06) | 1.56 (0.56, 4.36) 0.393 |  |
| PLT/WBC ratio | Low | 110 (2.16) | 1.64 (0.93, 2.89) 0.088 | 0.622 |
|  | Middle | 56 (1.10) | 1.70 (0.71, 4.08) 0.232 |  |
|  | High | 49 (0.96) | 2.40 (0.85, 6.78) 0.098 |  |

The multivariate model adjusted for age range, sex, race, smoking status, BMI, diabetes, hypertension, disseminated cancer, bleeding disorders, preoperative sepsis, emergency case, functional health status, ASA classification, BUN, AST, sodium, ALB, and PLT/WBC ratio, except for the variable that is stratified. BUN, AST, sodium, ALB, and PLT/WBC ratio were categorized into tertiles. ^*^The OR and 95% CI for the subgroup could not be reliably estimated due to the small number of events.

ALB, albumin; ASA, American Society of Anesthesiologists; AST, Aspartate Aminotransferase; BMI, body mass index; BUN, blood urea nitrogen; CI, confidence interval; OR, odds ratio; PE, pulmonary embolism; PLT/WBC, platelet-to-white blood cell.
