# Supplementary material for: Association between preoperative chronic steroid use and pulmonary embolism in adult tumor craniotomy: Linking role of platelet-to-white blood cell ratio
Source: PLoS One. 2026 Aug 4;21(8):e0355416. doi: 10.1371/journal.pone.0355416 (PMC13436738; doi:10.1371/journal.pone.0355416)
Supplement: Supplementary file 2 [file pone.0355416.s005.docx]

**S2 Table. Sensitivity analysis: mediation analysis of the role of PLT/WBC ratio in the association between preoperative chronic steroid use and postoperative PE across 50 multiple imputation datasets.**

| **MI Datasets** | ADE  Estimate (95% CI), P value | ACME  Estimate (95% CI), P value | Total effect  Estimate (95% CI), P value | Proportion mediated  Estimate (95% CI), P value |
| --- | --- | --- | --- | --- |
|  |  |  |  |  |
| MI Dataset 1 | 0.0094 (0.0030, 0.0164) 0.006 | 0.0010 (0.0002, 0.0021) 0.004 | 0.0104 (0.0039, 0.0177) 0.002 | 0.0941 (0.0209, 0.2750) 0.006 |
| MI Dataset 2 | 0.0095 (0.0029, 0.0167) 0.004 | 0.0010 (0.0002, 0.0021) 0.002 | 0.0105 (0.0039, 0.0177) 0.002 | 0.0965 (0.0260, 0.2746) 0.004 |
| MI Dataset 3 | 0.0095 (0.0030, 0.0166) 0.004 | 0.0009 (0.0002, 0.0020) 0.002 | 0.0104 (0.0039, 0.0180) 0.002 | 0.0892 (0.0214, 0.2578) 0.004 |
| MI Dataset 4 | 0.0094 (0.0028, 0.0163) 0.006 | 0.0009 (0.0002, 0.0019) 0.006 | 0.0103 (0.0039, 0.0176) 0.002 | 0.0890 (0.0200, 0.2691) 0.008 |
| MI Dataset 5 | 0.0091 (0.0028, 0.0160) 0.006 | 0.0008 (0.0001, 0.0018) 0.008 | 0.0099 (0.0036, 0.0172) 0.002 | 0.0857 (0.0149, 0.2615) 0.010 |
| MI Dataset 6 | 0.0094 (0.0030, 0.0169) 0.004 | 0.0009 (0.0002, 0.0019) 0.004 | 0.0103 (0.0039, 0.0178) 0.002 | 0.0872 (0.0184, 0.2575) 0.006 |
| MI Dataset 7 | 0.0096 (0.0030, 0.0169) 0.004 | 0.0010 (0.0003, 0.0021) 0.002 | 0.0106 (0.0041, 0.0181) 0.002 | 0.0936 (0.0264, 0.2654) 0.004 |
| MI Dataset 8 | 0.0093 (0.0028, 0.0166) 0.004 | 0.0009 (0.0002, 0.0020) 0.002 | 0.0103 (0.0038, 0.0177) 0.002 | 0.0922 (0.0244, 0.2652) 0.004 |
| MI Dataset 9 | 0.0096 (0.0031, 0.0169) 0.004 | 0.0010 (0.0002, 0.0021) 0.004 | 0.0106 (0.0040, 0.0180) 0.002 | 0.0928 (0.0226, 0.2726) 0.006 |
| MI Dataset 10 | 0.0093 (0.0030, 0.0165) 0.006 | 0.0009 (0.0002, 0.0020) 0.004 | 0.0102 (0.0038, 0.0176) 0.002 | 0.0908 (0.0205, 0.2746) 0.006 |
| MI Dataset 11 | 0.0095 (0.0031, 0.0167) 0.004 | 0.0009 (0.0002, 0.0020) 0.002 | 0.0105 (0.0040, 0.0178) 0.002 | 0.0893 (0.0207, 0.2599) 0.004 |
| MI Dataset 12 | 0.0093 (0.0029, 0.0163) 0.006 | 0.0009 (0.0001, 0.0019) 0.006 | 0.0102 (0.0039, 0.0174) 0.002 | 0.0856 (0.0156, 0.2570) 0.008 |
| MI Dataset 13 | 0.0095 (0.0030, 0.0168) 0.006 | 0.0009 (0.0002, 0.0019) 0.002 | 0.0105 (0.0040, 0.0180) 0.002 | 0.0878 (0.0238, 0.2515) 0.004 |
| MI Dataset 14 | 0.0089 (0.0025, 0.0159) 0.006 | 0.0008 (0.0002, 0.0017) 0.006 | 0.0097 (0.0034, 0.0168) 0.002 | 0.0804 (0.0163, 0.2548) 0.008 |
| MI Dataset 15 | 0.0092 (0.0027, 0.0162) 0.006 | 0.0009 (0.0002, 0.0019) 0.004 | 0.0101 (0.0036, 0.0173) 0.002 | 0.0867 (0.0197, 0.2637) 0.006 |
| MI Dataset 16 | 0.0093 (0.0028, 0.0166) 0.006 | 0.0009 (0.0002, 0.0019) 0.002 | 0.0102 (0.0037, 0.0176) 0.002 | 0.0867 (0.0179, 0.2579) 0.004 |
| MI Dataset 17 | 0.0093 (0.0028, 0.0164) 0.004 | 0.0010 (0.0002, 0.0022) 0.002 | 0.0103 (0.0038, 0.0177) 0.002 | 0.0951 (0.0226, 0.2811) 0.004 |
| MI Dataset 18 | 0.0090 (0.0027, 0.0159) 0.006 | 0.0009 (0.0002, 0.0020) 0.002 | 0.0099 (0.0036, 0.0171) 0.002 | 0.0928 (0.0194, 0.2800) 0.004 |
| MI Dataset 19 | 0.0091 (0.0027, 0.0160) 0.006 | 0.0009 (0.0002, 0.0020) 0.004 | 0.0101 (0.0036, 0.0172) 0.002 | 0.0919 (0.0211, 0.2720) 0.006 |
| MI Dataset 20 | 0.0093 (0.0028, 0.0164) 0.006 | 0.0010 (0.0002, 0.0020) 0.002 | 0.0102 (0.0038, 0.0174) 0.002 | 0.0931 (0.0243, 0.2689) 0.004 |
| MI Dataset 21 | 0.0094 (0.0029, 0.0167) 0.004 | 0.0009 (0.0002, 0.0020) 0.002 | 0.0104 (0.0037, 0.0178) 0.002 | 0.0900 (0.0219, 0.2646) 0.004 |
| MI Dataset 22 | 0.0094 (0.0030, 0.0167) 0.004 | 0.0010 (0.0002, 0.0021) 0.002 | 0.0104 (0.0040, 0.0179) 0.002 | 0.0946 (0.0263, 0.2721) 0.004 |
| MI Dataset 23 | 0.0095 (0.0029, 0.0167) 0.006 | 0.0010 (0.0002, 0.0021) 0.002 | 0.0105 (0.0039, 0.0179) 0.002 | 0.0942 (0.0228, 0.2716) 0.004 |
| MI Dataset 24 | 0.0091 (0.0027, 0.0163) 0.006 | 0.0008 (0.0002, 0.0018) 0.006 | 0.0100 (0.0036, 0.0171) 0.002 | 0.0830 (0.0160, 0.2602) 0.008 |
| MI Dataset 25 | 0.0096 (0.0030, 0.0169) 0.004 | 0.0009 (0.0002, 0.0020) 0.004 | 0.0105 (0.0038, 0.0180) 0.002 | 0.0891 (0.0220, 0.2539) 0.006 |
| MI Dataset 26 | 0.0094 (0.0029, 0.0165) 0.006 | 0.0009 (0.0002, 0.0019) 0.002 | 0.0103 (0.0038, 0.0176) 0.002 | 0.0870 (0.0204, 0.2602) 0.004 |
| MI Dataset 27 | 0.0093 (0.0029, 0.0164) 0.006 | 0.0009 (0.0002, 0.0019) 0.004 | 0.0102 (0.0037, 0.0175) 0.002 | 0.0841 (0.0195, 0.2509) 0.006 |
| MI Dataset 28 | 0.0091 (0.0027, 0.0159) 0.006 | 0.0008 (0.0001, 0.0018) 0.010 | 0.0099 (0.0035, 0.0171) 0.002 | 0.0822 (0.0157, 0.2525) 0.012 |
| MI Dataset 29 | 0.0095 (0.0030, 0.0167) 0.006 | 0.0009 (0.0002, 0.0018) 0.002 | 0.0103 (0.0039, 0.0178) 0.002 | 0.0831 (0.0204, 0.2390) 0.004 |
| MI Dataset 30 | 0.0094 (0.0029, 0.0163) 0.006 | 0.0009 (0.0002, 0.0019) 0.004 | 0.0103 (0.0038, 0.0174) 0.002 | 0.0873 (0.0213, 0.2581) 0.006 |
| MI Dataset 31 | 0.0095 (0.0029, 0.0166) 0.006 | 0.0009 (0.0002, 0.0019) 0.002 | 0.0104 (0.0038, 0.0176) 0.002 | 0.0874 (0.0221, 0.2508) 0.004 |
| MI Dataset 32 | 0.0093 (0.0029, 0.0162) 0.006 | 0.0009 (0.0002, 0.0020) 0.002 | 0.0102 (0.0038, 0.0176) 0.002 | 0.0922 (0.0212, 0.2751) 0.004 |
| MI Dataset 33 | 0.0094 (0.0028, 0.0165) 0.004 | 0.0009 (0.0002, 0.0019) 0.002 | 0.0103 (0.0039, 0.0178) 0.002 | 0.0896 (0.0220, 0.2601) 0.004 |
| MI Dataset 34 | 0.0092 (0.0029, 0.0163) 0.004 | 0.0009 (0.0002, 0.0020) 0.002 | 0.0101 (0.0037, 0.0176) 0.002 | 0.0897 (0.0211, 0.2625) 0.004 |
| MI Dataset 35 | 0.0093 (0.0029, 0.0164) 0.006 | 0.0009 (0.0002, 0.0019) 0.004 | 0.0102 (0.0037, 0.0174) 0.002 | 0.0861 (0.0187, 0.2579) 0.006 |
| MI Dataset 36 | 0.0090 (0.0027, 0.0161) 0.006 | 0.0008 (0.0002, 0.0019) 0.006 | 0.0099 (0.0036, 0.0171) 0.002 | 0.0857 (0.0177, 0.2595) 0.008 |
| MI Dataset 37 | 0.0093 (0.0028, 0.0163) 0.006 | 0.0009 (0.0002, 0.0019) 0.004 | 0.0101 (0.0037, 0.0173) 0.002 | 0.0867 (0.0171, 0.2614) 0.006 |
| MI Dataset 38 | 0.0093 (0.0027, 0.0162) 0.004 | 0.0009 (0.0002, 0.0020) 0.002 | 0.0102 (0.0038, 0.0176) 0.002 | 0.0920 (0.0218, 0.2713) 0.004 |
| MI Dataset 39 | 0.0092 (0.0027, 0.0162) 0.006 | 0.0009 (0.0002, 0.0019) 0.004 | 0.0102 (0.0037, 0.0175) 0.002 | 0.0917 (0.0234, 0.2726) 0.006 |
| MI Dataset 40 | 0.0092 (0.0027, 0.0162) 0.006 | 0.0009 (0.0002, 0.0020) 0.004 | 0.0101 (0.0037, 0.0175) 0.002 | 0.0898 (0.0211, 0.2692) 0.006 |
| MI Dataset 41 | 0.0094 (0.0029, 0.0165) 0.006 | 0.0009 (0.0002, 0.0019) 0.004 | 0.0103 (0.0038, 0.0177) 0.002 | 0.0877 (0.0223, 0.2584) 0.006 |
| MI Dataset 42 | 0.0093 (0.0028, 0.0163) 0.006 | 0.0009 (0.0002, 0.0019) 0.004 | 0.0102 (0.0037, 0.0175) 0.002 | 0.0882 (0.0204, 0.2578) 0.006 |
| MI Dataset 43 | 0.0096 (0.0031, 0.0169) 0.004 | 0.0010 (0.0002, 0.0020) 0.002 | 0.0106 (0.0041, 0.0180) 0.002 | 0.0917 (0.0257, 0.2644) 0.004 |
| MI Dataset 44 | 0.0091 (0.0027, 0.0161) 0.004 | 0.0009 (0.0002, 0.0018) 0.008 | 0.0100 (0.0035, 0.0173) 0.002 | 0.0855 (0.0191, 0.2608) 0.010 |
| MI Dataset 45 | 0.0096 (0.0030, 0.0168) 0.004 | 0.0010 (0.0002, 0.0020) 0.002 | 0.0106 (0.0041, 0.0182) 0.002 | 0.0904 (0.0227, 0.2582) 0.004 |
| MI Dataset 46 | 0.0092 (0.0027, 0.0162) 0.004 | 0.0009 (0.0002, 0.0019) 0.004 | 0.0101 (0.0036, 0.0174) 0.002 | 0.0876 (0.0188, 0.2658) 0.006 |
| MI Dataset 47 | 0.0096 (0.0031, 0.0170) 0.004 | 0.0010 (0.0003, 0.0022) 0.002 | 0.0107 (0.0043, 0.0183) 0.002 | 0.0970 (0.0264, 0.2819) 0.004 |
| MI Dataset 48 | 0.0095 (0.0031, 0.0169) 0.004 | 0.0010 (0.0003, 0.0022) 0.002 | 0.0105 (0.0040, 0.0179) 0.002 | 0.0979 (0.0258, 0.2763) 0.004 |
| MI Dataset 49 | 0.0094 (0.0029, 0.0165) 0.006 | 0.0010 (0.0002, 0.0021) 0.002 | 0.0103 (0.0039, 0.0176) 0.002 | 0.0938 (0.0234, 0.2746) 0.004 |
| MI Dataset 50 | 0.0093 (0.0029, 0.0164) 0.004 | 0.0010 (0.0002, 0.0021) 0.004 | 0.0103 (0.0038, 0.0177) 0.002 | 0.0933 (0.0217, 0.2710) 0.006 |
| Pool estimates | 0.0093 (0.0025, 0.0162) 0.007 | 0.0009 (0.0000, 0.0018)^*^ 0.043 | 0.0103 (0.0033, 0.0172) 0.004 | 0.0895 (0.0216, 0.3050) 0.002 |

^*^ Unrounded 95% CI: (0.00002968731, 0.001810153). Adjusted for age range, sex, race, smoking status, body mass index, diabetes, hypertension, disseminated cancer, bleeding disorders, preoperative sepsis, emergency case, functional health status, American Society of Anesthesiologists classification, blood urea nitrogen, aspartate aminotransferase, sodium, and albumin (n = 15,274; 215 PE events). Estimates and 95% confidence intervals were calculated using the nonparametric bootstrap method with 1,000 resamples.

ACME, average causal mediation effect; ADE, average direct effect; MI, multiple imputation; PE, pulmonary embolism; PLT/WBC, platelet-to-white blood cell ratio.
