# Supplementary material for: Association between preoperative chronic steroid use and pulmonary embolism in adult tumor craniotomy: Linking role of platelet-to-white blood cell ratio
Source: PLoS One. 2026 Aug 4;21(8):e0355416. doi: 10.1371/journal.pone.0355416 (PMC13436738; doi:10.1371/journal.pone.0355416)
Supplement: Supplementary file 4 [file pone.0355416.s007.docx]

**S4 Table. Sensitivity analysis: comparison of characteristics between the included and excluded patients.**

| **Variables** | **Excluded patients (n = 3368)** | **Included patients (n = 15274)** | P value |
| --- | --- | --- | --- |
| Preoperative chronic steroid use |  |  |  |
| Non-steroid | 2888 (85.75%) | 12955 (84.82%) | 0.171 |
| Chronic steroid | 480 (14.25%) | 2319 (15.18%) |  |
| Age range, n (%) |  |  | <0.001 |
| 18-40 years | 660 (19.60%) | 2397 (15.69%) |  |
| 41-60 years | 1423 (42.25%) | 6319 (41.37%) |  |
| 61-80 years | 1187 (35.24%) | 6046 (39.58%) |  |
| ≥ 81 years | 98 (2.91%) | 512 (3.35%) |  |
| Sex, n (%) |  |  | <0.001 |
| Female | 1911 (56.74%) | 7895 (51.69%) |  |
| Male | 1457 (43.26%) | 7379 (48.31%) |  |
| Race, n (%) |  |  | <0.001 |
| White | 2507 (74.44%) | 10783 (70.60%) |  |
| Black | 211 (6.26%) | 1034 (6.77%) |  |
| Other | 650 (19.30%) | 3457 (22.63%) |  |
| Smoking status, n (%) |  |  | <0.001 |
| No | 2839 (84.29%) | 12193 (79.83%) |  |
| Yes | 529 (15.71%) | 3081 (20.17%) |  |
| BMI, n (%) |  |  | <0.001 |
| < 25.0 kg/m^2^ | 914 (27.37%) | 4599 (31.56%) |  |
| 25.0-29.9 kg/m^2^ | 1156 (34.61%) | 4945 (33.93%) |  |
| ≥ 30.0 kg/m^2^ | 1270 (38.02%) | 5028 (34.50%) |  |
| Diabetes, n (%) |  |  | 0.077 |
| No | 3004 (89.19%) | 13458 (88.11%) |  |
| Yes | 364 (10.81%) | 1816 (11.89%) |  |
| Hypertension, n (%) |  |  | 0.151 |
| No | 2119 (62.92%) | 9407 (61.59%) |  |
| Yes | 1249 (37.08%) | 5867 (38.41%) |  |
| Disseminated cancer, n (%) |  |  | <0.001 |
| No | 2994 (88.90%) | 11626 (76.12%) |  |
| Yes | 374 (11.10%) | 3648 (23.88%) |  |
| Bleeding disorders, n (%) |  |  | <0.001 |
| No | 3334 (98.99%) | 14935 (97.78%) |  |
| Yes | 34 (1.01%) | 339 (2.22%) |  |
| Preoperative sepsis, n (%) |  |  | <0.001 |
| No | 3356 (99.64%) | 14614 (95.68%) |  |
| Yes | 12 (0.36%) | 660 (4.32%) |  |
| Emergency case, n (%) |  |  | <0.001 |
| No | 3341 (99.20%) | 14103 (92.33%) |  |
| Yes | 27 (0.80%) | 1171 (7.67%) |  |
| Functional health status, n (%) |  |  | <0.001 |
| Independent | 3261 (97.52%) | 14525 (95.51%) |  |
| Dependent | 83 (2.48%) | 683 (4.49%) |  |
| ASA classification, n (%) |  |  | <0.001 |
| Class I-II | 1294 (38.59%) | 3736 (24.70%) |  |
| Class III-V | 2059 (61.41%) | 11387 (75.30%) |  |
| BUN, mg/dL, median (Q1-Q3) | 15.00 (12.00-19.00) | 16.00 (12.00-21.00) | <0.001 |
| AST, U/L, median (Q1-Q3) | 21.00 (17.00-27.00) | 21.00 (16.00-28.00) | 0.654 |
| Sodium, mmol/L, mean ± SD | 139.26 ± 3.00 | 138.51 ± 3.25 | <0.001 |
| ALB, g/dL, mean ± SD | 4.10 ± 0.47 | 3.86 ± 0.57 | <0.001 |
| PLT/WBC ratio, median (Q1-Q3) | 32.75 (25.45-41.33) | 26.76 (19.28-36.03) | <0.001 |
| PE |  |  | 0.123 |
| No | 3332 (98.93%) | 15059 (98.59%) |  |
| Yes | 36 (1.07%) | 215 (1.41%) |  |

ALB, albumin; ASA, American Society of Anesthesiologists; AST, Aspartate Aminotransferase; BMI, body mass index; BUN, blood urea nitrogen; PE, pulmonary embolism; PLT/WBC, platelet-to-white blood cell; SD, standard deviation.
