# Supplementary material for: Association between preoperative chronic steroid use and pulmonary embolism in adult tumor craniotomy: Linking role of platelet-to-white blood cell ratio
Source: PLoS One. 2026 Aug 4;21(8):e0355416. doi: 10.1371/journal.pone.0355416 (PMC13436738; doi:10.1371/journal.pone.0355416)
Supplement: Supplementary file 6 [file pone.0355416.s009.docx]

**S6 Table. Sensitivity analysis: comparison of characteristics between participants with complete ALB and AST data and those with missing ALB or AST data.**

| **Variables** | **Participants with missing ALB or AST (n = 7580)** | **Participants with complete ALB and AST (n = 7694)** | P value |
| --- | --- | --- | --- |
| Preoperative chronic steroid use |  |  |  |
| Non-steroid | 6555 (86.48%) | 6400 (83.18%) | <0.001 |
| Chronic steroid | 1025 (13.52%) | 1294 (16.82%) |  |
| Age range, n (%) |  |  | <0.001 |
| 18-40 years | 1366 (18.02%) | 1031 (13.40%) |  |
| 41-60 years | 3107 (40.99%) | 3212 (41.75%) |  |
| 61-80 years | 2852 (37.63%) | 3194 (41.51%) |  |
| ≥ 81 years | 255 (3.36%) | 257 (3.34%) |  |
| Sex, n (%) |  |  | <0.001 |
| Female | 4117 (54.31%) | 3778 (49.10%) |  |
| Male | 3463 (45.69%) | 3916 (50.90%) |  |
| Race, n (%) |  |  | <0.001 |
| White | 4977 (65.66%) | 5806 (75.46%) |  |
| Black | 420 (5.54%) | 614 (7.98%) |  |
| Other | 2183 (28.80%) | 1274 (16.56%) |  |
| Smoking status, n (%) |  |  | <0.001 |
| No | 6194 (81.72%) | 5999 (77.97%) |  |
| Yes | 1386 (18.28%) | 1695 (22.03%) |  |
| BMI, n (%) |  |  | 0.081 |
| < 25.0 kg/m^2^ | 2172 (30.67%) | 2427 (32.40%) |  |
| 25.0-29.9 kg/m^2^ | 2435 (34.39%) | 2510 (33.51%) |  |
| ≥ 30.0 kg/m^2^ | 2474 (34.94%) | 2554 (34.09%) |  |
| Diabetes, n (%) |  |  | <0.001 |
| No | 6754 (89.10%) | 6704 (87.13%) |  |
| Yes | 826 (10.90%) | 990 (12.87%) |  |
| Hypertension, n (%) |  |  | <0.001 |
| No | 4860 (64.12%) | 4547 (59.10%) |  |
| Yes | 2720 (35.88%) | 3147 (40.90%) |  |
| Disseminated cancer, n (%) |  |  | <0.001 |
| No | 6305 (83.18%) | 5321 (69.16%) |  |
| Yes | 1275 (16.82%) | 2373 (30.84%) |  |
| Bleeding disorders, n (%) |  |  | 0.002 |
| No | 7440 (98.15%) | 7495 (97.41%) |  |
| Yes | 140 (1.85%) | 199 (2.59%) |  |
| Preoperative sepsis, n (%) |  |  | <0.001 |
| No | 7379 (97.35%) | 7235 (94.03%) |  |
| Yes | 201 (2.65%) | 459 (5.97%) |  |
| Emergency case, n (%) |  |  | 0.590 |
| No | 6990 (92.22%) | 7113 (92.45%) |  |
| Yes | 590 (7.78%) | 581 (7.55%) |  |
| Functional health status, n (%) |  |  | <0.001 |
| Independent | 7288 (96.48%) | 7237 (94.55%) |  |
| Dependent | 266 (3.52%) | 417 (5.45%) |  |
| ASA classification, n (%) |  |  | <0.001 |
| Class I-II | 2339 (31.23%) | 1397 (18.30%) |  |
| Class III-V | 5151 (68.77%) | 6236 (81.70%) |  |
| BUN, mg/dL, median (Q1-Q3) | 16.00 (12.00-20.00) | 17.00 (13.00-22.00) | <0.001 |
| Sodium, mmol/L, mean ± SD | 138.58 ± 3.14 | 138.43 ± 3.36 | 0.005 |
| PLT/WBC ratio, median (Q1-Q3) | 27.87 (20.29-37.14) | 25.66 (18.53-34.49) | <0.001 |
| PE |  |  | 0.031 |
| No | 7489 (98.80%) | 7570 (98.39%) |  |
| Yes | 91 (1.20%) | 124 (1.61%) |  |

ALB, albumin; ASA, American Society of Anesthesiologists; AST, Aspartate Aminotransferase; BMI, body mass index; BUN, blood urea nitrogen; PE, pulmonary embolism; PLT/WBC, platelet-to-white blood cell; SD, standard deviation.
