# Supplementary material for: Association between preoperative chronic steroid use and pulmonary embolism in adult tumor craniotomy: Linking role of platelet-to-white blood cell ratio
Source: PLoS One. 2026 Aug 4;21(8):e0355416. doi: 10.1371/journal.pone.0355416 (PMC13436738; doi:10.1371/journal.pone.0355416)
Supplement: Supplementary file 7 [file pone.0355416.s010.docx]

**S7 Table. Sensitivity analysis: the association between preoperative chronic steroid use and postoperative PE among participants with complete ALB and AST data.**

| **Exposure** | **Crude model** | | **Model 1** | | **Model 3** | |
| --- | --- | --- | --- | --- | --- | --- |
|  | **OR (95% CI)** | ***P* value** | **OR (95% CI)** | ***P* value** | **OR (95% CI)** | ***P* value** |
| Non-steroid | 1.00 (Reference) |  | 1.00 (Reference) |  | 1.00 (Reference) |  |
| Chronic steroid | 2.05 (1.39, 3.04) | < 0.001 | 2.07 (1.39, 3.06) | < 0.001 | 2.01 (1.32, 3.07) | 0.001 |

Model 1: adjusted for age range, sex and race (n = 7,694; 124 PE events); Model 3: adjusted for age range, sex, race, smoking status, body mass index, diabetes, hypertension, disseminated cancer, bleeding disorders, preoperative sepsis, emergency case, functional health status, American Society of Anesthesiologists classification, blood urea nitrogen, and sodium (n = 7,360; 115 PE events).

ALB, albumin; AST, Aspartate Aminotransferase; CI, confidence interval; OR, odds ratio; PE, pulmonary embolism.
