# Supplementary material for: Association between preoperative chronic steroid use and pulmonary embolism in adult tumor craniotomy: Linking role of platelet-to-white blood cell ratio
Source: PLoS One. 2026 Aug 4;21(8):e0355416. doi: 10.1371/journal.pone.0355416 (PMC13436738; doi:10.1371/journal.pone.0355416)
Supplement: Supplementary file 8 [file pone.0355416.s011.docx]

**S8 Table. Sensitivity analysis: mediation analysis of the role of PLT/WBC ratio in the association between preoperative chronic steroid use and postoperative PE among participants with complete ALB and AST data.**

| **Analysis** | **Estimate** | **95% CI** | P value |
| --- | --- | --- | --- |
| ADE | 0.0119 | (0.0032, 0.0209) | 0.008 |
| ACME | 0.0019 | (0.0008, 0.0033) | <0.001 |
| Total effect | 0.0137 | (0.0049, 0.0233) | 0.002 |
| Proportion of mediation (%) | 13.58 | (5.95, 37.90) | 0.002 |

Adjusted for age range, sex, race, smoking status, body mass index, diabetes, hypertension, disseminated cancer, bleeding disorders, preoperative sepsis, emergency case, functional health status, American Society of Anesthesiologists classification, blood urea nitrogen, and sodium (n = 7,360; 115 PE events). Estimates and 95% confidence intervals were calculated using the nonparametric bootstrap method with 1,000 resamples.

ACME, average causal mediation effect; ADE, average direct effect; ALB, albumin; AST, Aspartate Aminotransferase; PE, pulmonary embolism; PLT/WBC, platelet-to-white blood cell.
