# Supplementary material for: Association between preoperative chronic steroid use and pulmonary embolism in adult tumor craniotomy: Linking role of platelet-to-white blood cell ratio
Source: PLoS One. 2026 Aug 4;21(8):e0355416. doi: 10.1371/journal.pone.0355416 (PMC13436738; doi:10.1371/journal.pone.0355416)
Supplement: Supplementary file 11 [file pone.0355416.s014.docx]

**S11 Table. Sensitivity analysis: mediation analysis of the role of PLT count in the association between preoperative chronic steroid use and postoperative PE.**

| **Analysis** | **Estimate** | **95% CI** | P value |
| --- | --- | --- | --- |
| ADE | 0.0115 | (0.0028, 0.0205) | 0.008 |
| ACME | 0.0006 | (-0.0001, 0.0015) | 0.088 |
| Total effect | 0.0121 | (0.0036, 0.0209) | 0.006 |
| Proportion of mediation (%) | 5.06 | (-0.93, 19.38) | 0.090 |

Adjusted for age range, sex, race, smoking status, body mass index, diabetes, hypertension, disseminated cancer, bleeding disorders, preoperative sepsis, emergency case, functional health status, American Society of Anesthesiologists classification, blood urea nitrogen, aspartate aminotransferase, sodium, and albumin (n = 7,360; 115 PE events). Estimates and 95% confidence intervals were calculated using the nonparametric bootstrap method with 1,000 resamples.

ACME, average causal mediation effect; ADE, average direct effect; PE, pulmonary embolism; PLT, platelet.
